# Biomechanical analysis of bridge combined fixation system as a novel treatment for the fixation of type A3 distal femoral fractures

**DOI:** 10.3389/fsurg.2023.1264904

**Published:** 2023-11-16

**Authors:** Jianke Liu, Zhaozhao Huang, Yubin Qi, Yuntao Long, Yanhui Zhang, Na Liu, Guilai Zuo, Wen Wang

**Affiliations:** ^1^ Shandong First Medical University & Shandong Academy Medical Sciences, Jinan, China; ^2^ Tianjin Walkman Biomaterial Co., Ltd. Newton Laboratory, Tianjin, China; ^3^ Department of Orthopaedics, The First Affiliated Hospital of Shandong First Medical University & Shandong Provincial Qianfoshan Hospital, Jinan, China

**Keywords:** biomechanics, type A3 distal femoral fractures, bridge combined fixation system, locking compression plate, locking reconstruction plate

## Abstract

**Background:**

To compare the biomechanical parameters of AO/OTA type A3 distal femoral fractures fixed bilaterally with a bridge combined fixation system (BCFS) and lateral locking compression plate + locking reconstruction plate (LCP + LRP).

**Methods:**

Twelve A3 distal femoral fracture models with medial cortical defects of the distal femur were created using synthetic femoral Sawbones. BCFS and LCP + LRP were used for bilateral fixation, with six in each group. Axial compression and torsion tests were performed on the two groups of fracture models to determine their stiffness during axial compression and the Torsional stiffness during torsion tests. Axial compression failure tests were performed to collect the vertical loads of the ultimate failure tests.

**Results:**

In the test conducted on the fixed type A3 distal femoral fracture models, the axial stiffness in the BCFS group (group A) (1,072.61 ± 113.5 N/mm) was not significantly different from that in the LCP + LRP group (group B) (1,184.13 ± 110.24 N/mm) (t = 1.726, P = 0.115), the Torsional stiffness in group A (3.73 ± 0.12 N.m/deg) was higher than that in group B (3.37 ± 0.04 N.m/deg) (*t* = 6.825, *P* < 0.001),and the ultimate failure test of type A3 fracture model showed that the vertical load to destroy group A fixation (5,290.45 ± 109.63 N) was higher than that for group B (3,978.43 ± 17.1 N) (*t* = 23.28, *P* < 0.05). Notably, intertrochanteric fractures occurred in groups A and B.

**Conclusions:**

In the fixation of type A3 distal femoral fractures, the anti-axial compression of the BCFS group was similar to that of the LCP + LRP group, but the anti-torsion was better.

## Background

Supracondylar fractures of the femur within 15 cm of the medial and lateral femoral condyles, femoral intercondylar, and knee surfaces are collectively referred to as distal femoral fractures (1). They account for approximately 6% of femoral fractures in adults (2) and are relatively complex types of femoral fractures. Muller's AO/OTA classification is currently used in classifying distal femoral fractures, and A3 fractures with medial cortical defects are gradually increasing for several reasons, such as osteoporosis.

Type A3 fractures with medial cortical defects have the following characteristics: gradual widening of the medullary cavity from the middle femur, gradual thinning of the cortical bone, and cancellous bone replacement from the medullary cavity to the distal femur, which can easily affect the strength of the internal fracture fixation (3). After fracturing at this site, there is a high incidence of complications, such as varus deformity, nonunion, and internal fixation failure (4). Previous studies have suggested that lack of support for comminution of the medial cortex may be one of the important causes of these complications (5, 6). Currently, the most commonly used treatment methods for type A3 fractures of the distal femur are divided into two, namely, extramedullary fixation and intramedullary fixation methods (7). Intramedullary fixation causes several problems, such as postoperative loss of knee flexion, loss of reduction, screw breakage, nail extension into the joint, and knee pain (8). However, the application of an extramedullary locking plate has significantly improved the surgical results of middle and distal femoral fractures (9–11), and it has become the first choice for the treatment of type A3 fractures with medial cortical defects (12). Extramedullary fixation can be further divided into two methods: unilateral plate fixation and bilateral plate fixation. However, studies have shown that the incidence of nonunion of distal femoral fractures treated with a lateral locking plate alone ranges from 0% to 10% (13, 14), which may be related to the insufficient stability of single plate eccentric fixation and the type of fracture (15, 16). Therefore, some scholars believe that it is more feasible to select double plates as a treatment for distal femoral fractures with medial cortical defects (17–20), as the placement of a medial plate improves the overall strength of the internal fixation and strengthens resistance to deformation. However, the double plate still has defects, including large incision exposure during placement, resulting in prolonged operative time, increased bleeding, and difficulty placing staples medially (21).

**Figure 1:**
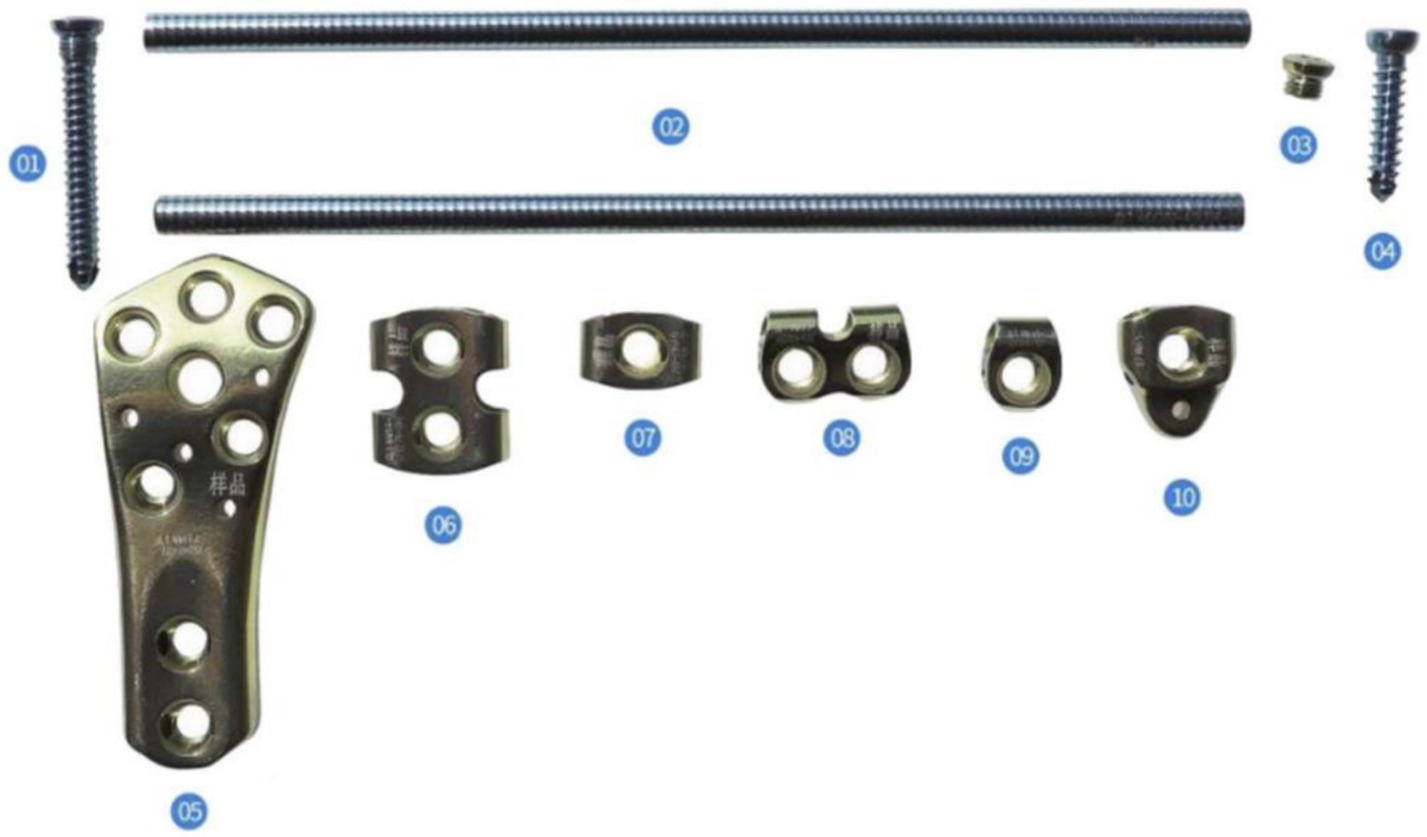
Basic unit components of the BCFS. (1) Locking screws, (2) connecting rod, (3) locking nut, (4) ordinary screws, (5) distal-shaped piece of the femur, (6) double-rod double-hole fixing block, (7) double-rod single-hole fixing block, (8) single-rod double-hole fixing block, (9) single-rod single-hole fixing block, and (10) end block fixing block.

In 2019, Kang et al. (22) and Wang et al. (23) used a bridge combined fixation system (BCFS) to treat distal femoral fractures in the elderly and achieved satisfactory results. The BCFS consists of metal connecting rods, locking screws, fixation blocks, and common screws, As shown in Figure 1, the fixation block placement direction and corresponding angle in BCFS can be adjusted in multiple directions so that easier and more flexible screw placement can be achieved. Furthermore, more flexible bicortical channel fixation can be achieved with less trauma around the medial and lateral femoral condyles than that when using the single-plate system. However, because the theoretical system of BCFS biomechanical basic research is not perfect, its application and promotion in the treatment of type A3 fractures are limited.

The distal femoral bridging modular internal fixation system (as shown in Figure 1) is mainly composed of distal-shaped piece of the femur (05), fixing block (including double-rod single-hole fixing block (07) and single-rod single-hole fixing block (09)), connecting rod (02), locking screws (01) and locking nut (03). Connecting rod (02) in BCFS with longitudinal spacer slot design. The attachment surface of the distal-shaped piece of the femur (05) conforms to the anatomical shape of the lateral femoral condyle, and its end is provided with two connecting rod holes and two locking screw holes, the rod fixation block (07, 09) is internally provided with a connecting rod hole parallel to the main plane of the fixation block, a screw hole is provided perpendicular to the main plane of the fixation block, and the connecting rod hole is locally crossed with the screw hole. Through the distal femoral anatomical block (07), the rod fixation block (07, 09) is matched with the locking nut (03) or locking screws (01), the distal femoral anatomical block and the rod fixation block are tightly pressed on the connecting rod, greatly improving the friction force and preventing loosening and slippage. According to the mechanical mechanical principle, this internal fixation device makes full use of the inherent mechanical rigidity of fixation block, connecting rod, common screw and locking screw as well as the free structure, length and angle combination between each other to form a rigid combined fixation structure, reduces and fixes the fracture bone block into the original physiological overall structure state, overcomes the improper stress generated by the traditional fixation device itself and the adverse consequences caused by the pressure on the periosteum, improves the reduction effect, expands the scope of indications and reduces the occurrence of complications.

Therefore, to verify the biomechanical reliability of BCFS in the treatment of type A3 distal femoral fractures, we designed an BCFS bilateral fixation distal femoral AO/OTA classification type A3 fracture model using the biomechanical internal fixation concept of LCP + LRP bilateral fixation system in conjunction with the advantages of BCFS products and performed a comparative biomechanical study with LCP + LRP bilateral fixation distal femoral AO/OTA classification type A3 fracture model. The objectives of this study include the following: (1) to compare the biomechanical differences between BCFS and LCP + LRP in bilateral fixation of AO/OTA classification type A3 distal femoral fractures, (2) to introduce the characteristics of the BCFS system, and (3) to elucidate the advantages of the BCFS system in the treatment of type A3 distal femoral fractures.

## Methods

### Test materials

Sawbones (third-generation composite femur, medium size 3,304; Pacific Research Laboratory, Vashon, WA, USA) in normal bone; Bridging Modular Fixation System: 6 mm diameter rod, block, and set screw (Tianjin Weiman Biomaterials Co., Ltd., China); Lateral distal femoral locking compression plate (Type I) (185 mm × 6 holes left); Locking Straight Reconstruction Plate (144 mm × 12 holes).

### Model construction

Standard gap osteotomy was performed on Sawbones (24); a standard-sized medial wedge was removed by a standard serrated cut starting 6 cm from the lateral joint line of the distal metaphysis, resulting in a medial cortical defect of 1 cm. The developed model was used to simulate AO/OTA type A3 distal femoral fractures that had lost medial support. The prepared distal femoral fracture models were treated with a lateral locking compression plate of the distal femur, a locking straight reconstruction plate, and a bridging combined fixation system for bilateral internal fixation. The length of the lateral BCFS rod combined with the distal femoral heterotypic block was consistent with that of the lateral locking compression plate of the distal femur (Type I), and the length of the medial BCFS rod was consistent with that of the 12-hole locking straight reconstruction plate. The screw placement position was consistent in the two groups, and bicortical fixation was performed by placing the distal femur in the planned position.

**Figure 2:**
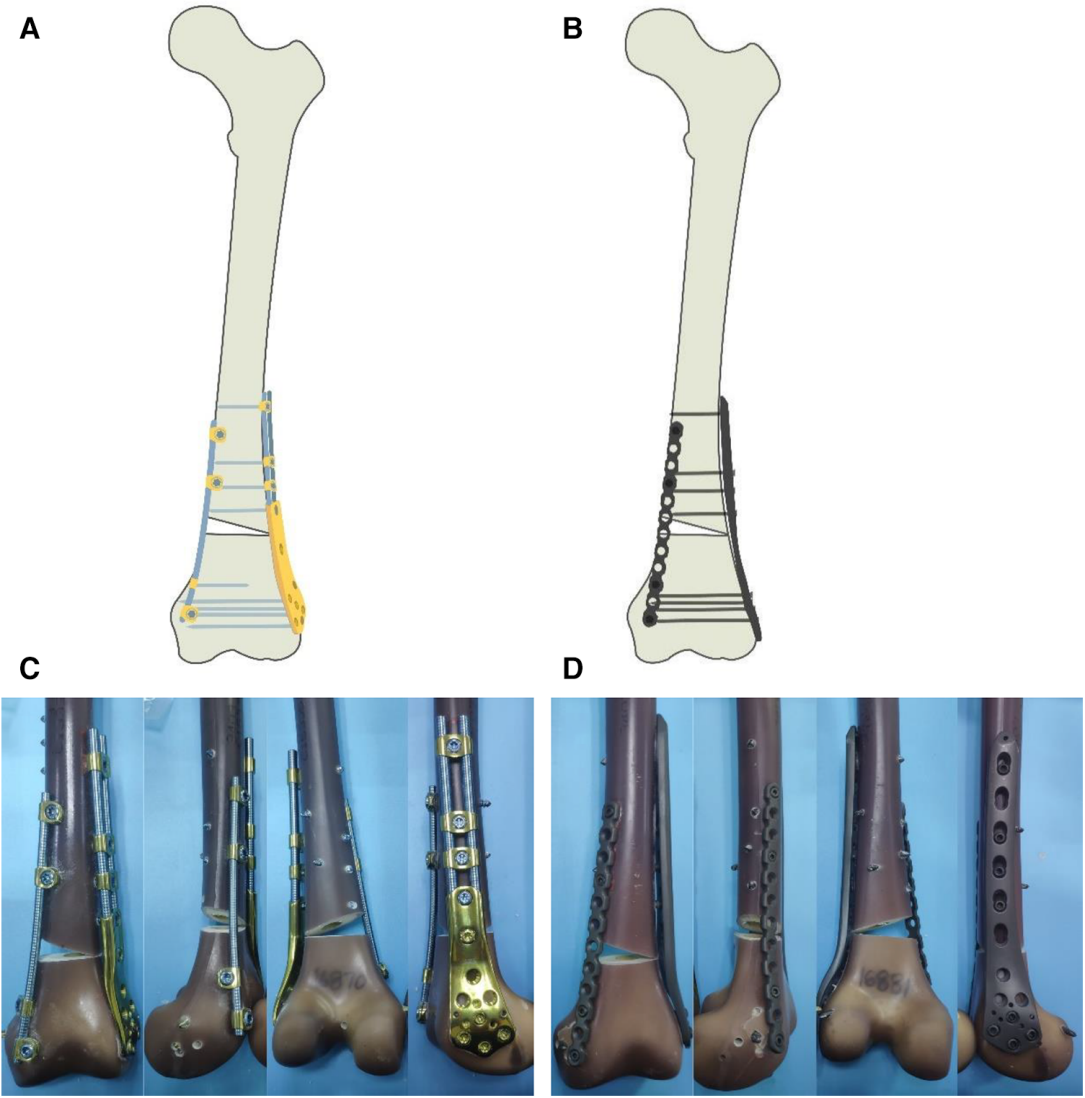
(**A**) model of AO/OTA classification A3 fracture of distal femur fixed bilaterally with BCFS, (**B**) model of AO/OTA classification A3 fracture of distal femur fixed bilaterally with LCP + LRP, (**C**) BCFS group, (**D**) LCP + LRP group.

BCFS Group (Group A): (a) Lateral distal femur: Two connecting rods, 12 cm in length and 6 mm in diameter, were selected and used in combination with heterotypic blocks of the distal femur. The fracture line was fixed proximally with four locking screws. Distal femoral heterotypic blocks were fixed bicortically using four locking screws at the distal end of the fracture. (b) Medial distal femur: A connecting rod with a length of 14.4 cm and diameter of 5 mm was used to perform bicortical fixation from the proximal to distal fracture with four single-rod single-hole fixation blocks, including two pieces proximal to the fracture line and two pieces distal to the fracture line, as shown in Figures 2A,C.

LCP + LRP group (Group B): (a) Lateral distal femur: Lateral distal femur locking compression plate and corresponding locking screws were selected, and four 5.0 mm diameter locking screws were used to fix the compression plate proximally to the fracture line, and bicortical locking screw fixation was performed. Likewise, four compression plates with 5 mm diameter screws were used to fix the distal end of the fracture line for bicortical fixation. (b) Medial distal femur: A locking straight reconstruction plate and corresponding screws were selected, and two 5.0 mm diameter locking screw fixation reconstruction plates were used each for bicortical fixation at the proximal end of the fracture plates and unicortical locking screw fixation at the distal end, as shown in Figures 2B,D.

### Experimental grouping

The method of Long et al. (25) and other tests and statistical analyses were adopted to determine the number of test models. Similarly, to simulate the type A3 distal femur fractures with medial cortical defects in humans, bilateral fixation fracture models were created using BCFS and LCP + LRP. Two groups, six in each, including group A for the BCFS fixation group and group B for the LCP + LRP fixation group, were created, and this grouping protocol is applicable to the full text.

### Biomechanical tests

**Figure 3:**
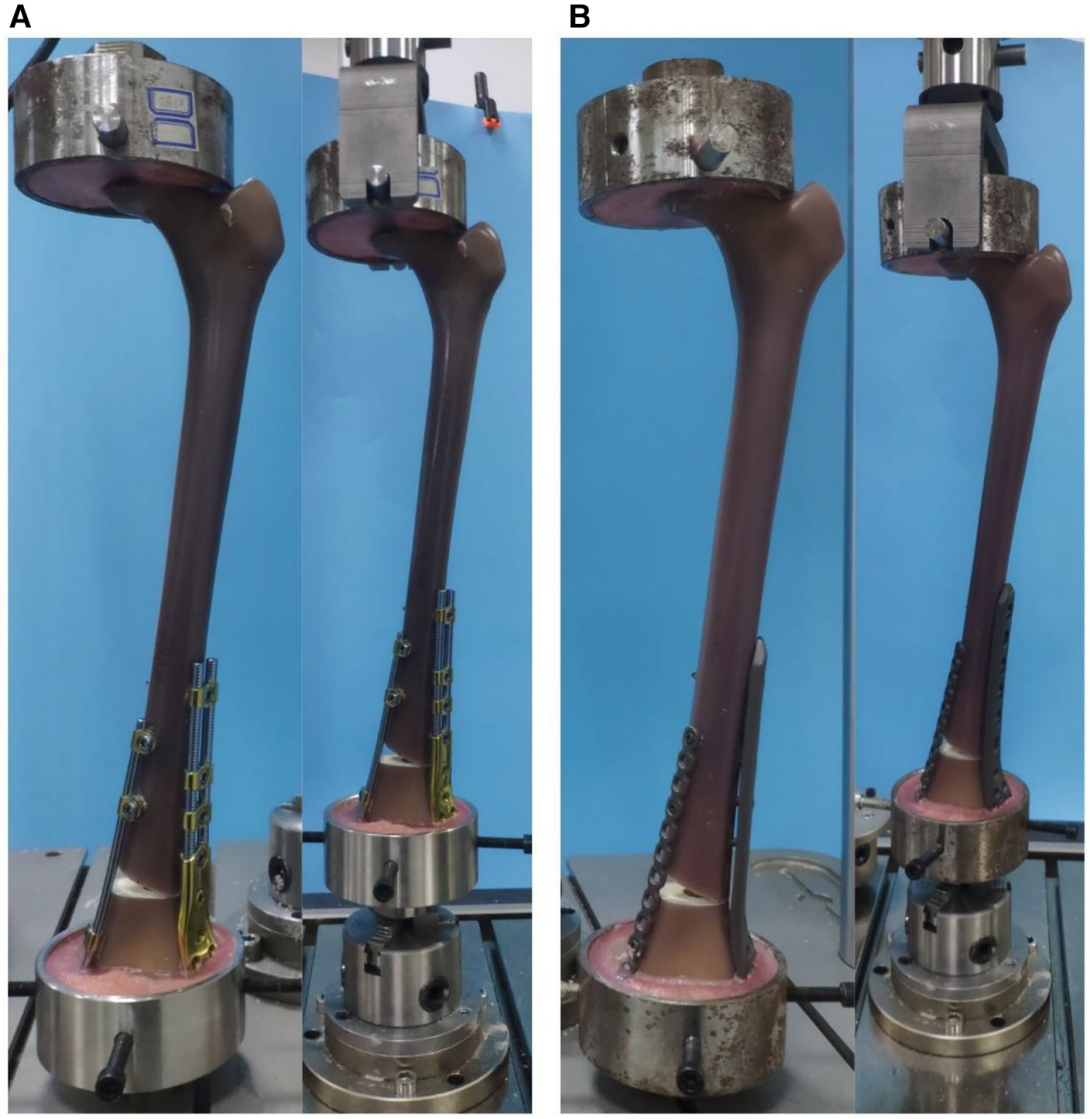
(**A**) BCFS model on an experimental machine, (**B**) LCP + LRP model on an experimental machine.

The models were fixed in the said fixation mode, and axial compression and torsion tests were conducted on all samples. Axial compression failure was also performed on all sample models to obtain their corresponding axial failure load, i.e., the load that resulted in irreversible failure of the implant or femur, as shown in Figures 3A,B.

#### Axial compression test

**Figure 4:**
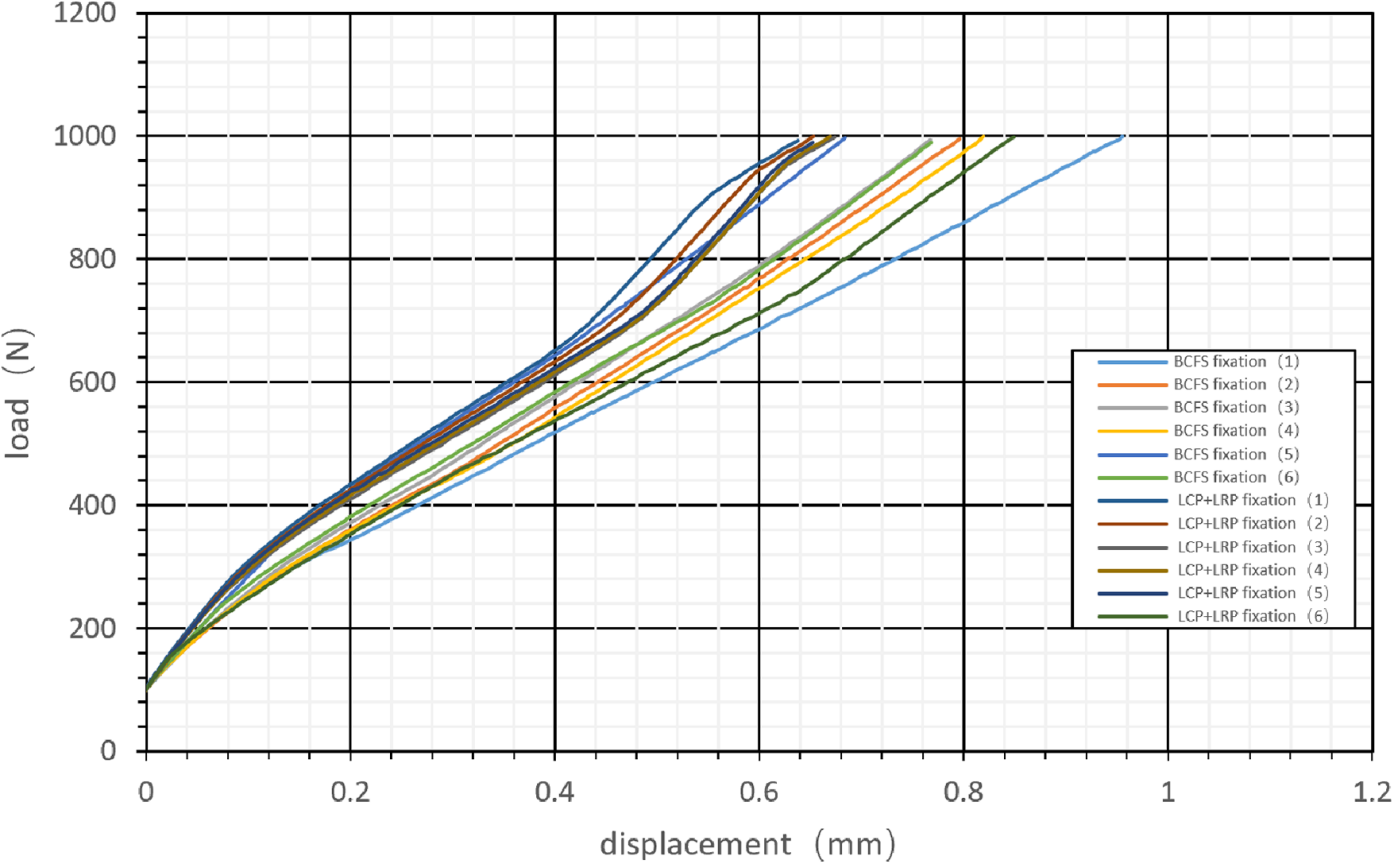
Load–displacement curves obtained by applying a vertical load of 100–1,000 N to the femoral head and performing the test at a displacement loading rate of 5 mm/min.

In carrying out this test, we used an Instron tensile fatigue tester (E10000) [General Standard Technical Services (Tianjin) Co., Ltd., Tianjin, China]. Iron cups were placed on the proximal and distal femur heads to fit the testing machine. A vertical load of 100–1,000 N attached to the proximal femur (equivalent to the physiological load of daily static activities) was tested at a displacement loading rate of 5 mm/min (26), and the load-displacement curve was obtained (Figure 4). Bluehill 2 software (Instron Corporation, UK) was then used to obtain the slope of the curve (i.e., the stiffness value).

#### Torsion test

**Figure 5:**
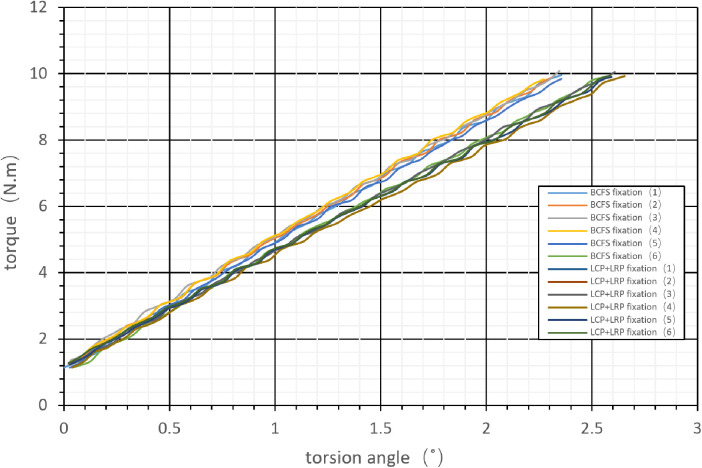
Torsional load applied to both ends of the femur; the maximum value is set as 10 Nm, and the loading rate is controlled at 25°/min to obtain the torque-torsion angle curve.

A torsion testing machine (ND-200, Changchun New Testing Instrument Co., Ltd., China) was used, and the femur was mounted to the equipment horizontally. Iron cups were placed proximally and distally to match and fit the testing machine. Torsional loads were applied to both ends of the femur with the maximum value set at 10 N m, and the loading rate was controlled at 25°/min to conduct the test to obtain the torque-torsion angle curve (Figure 5). The torsion angle data were obtained using the *P* main 1.0 software (Changchun Kexin Testing Instrument Co., Ltd., China).

#### Axial compression failure test

**Figure 6:**
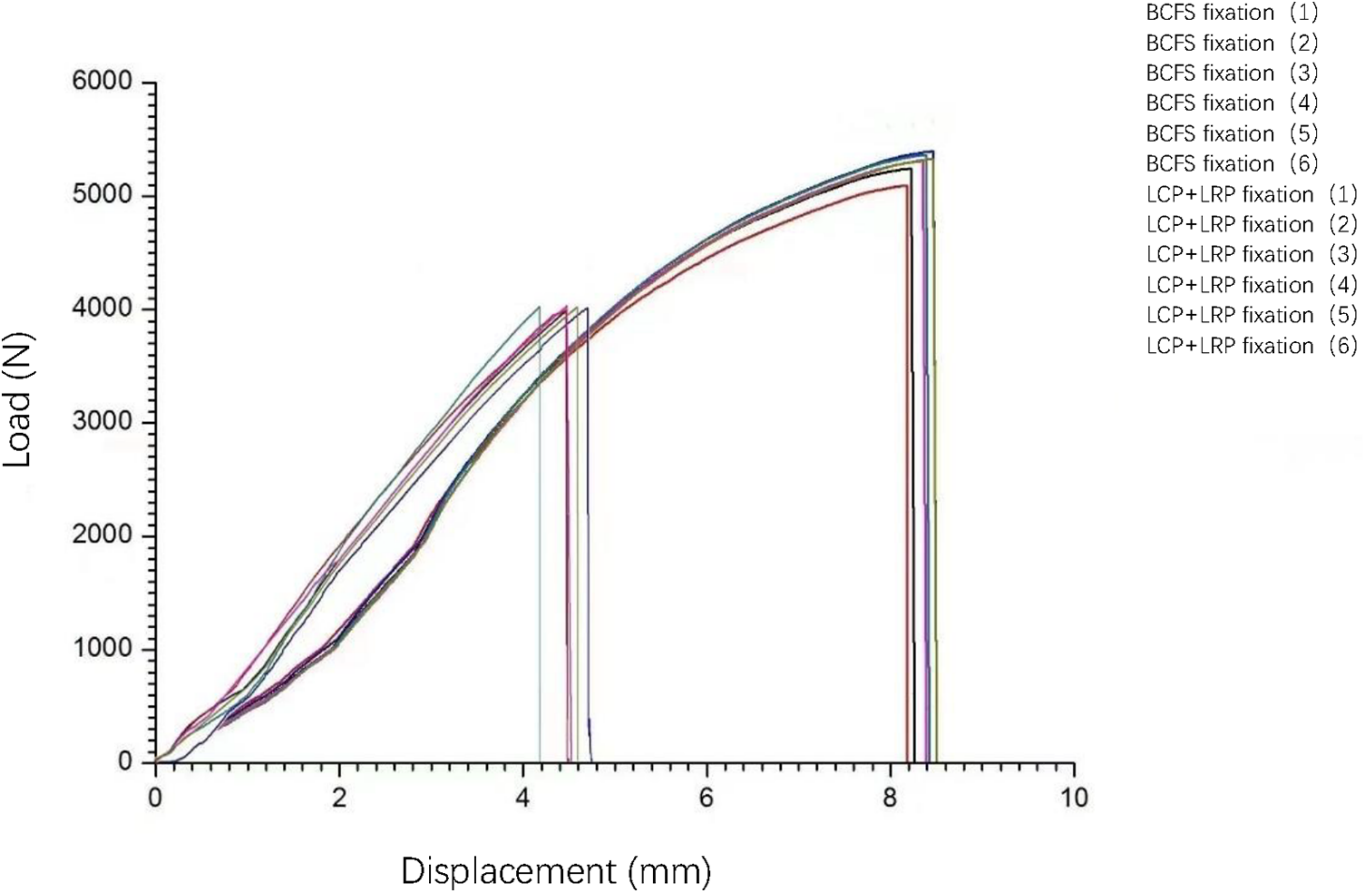
Test using an initial load of 100 N and displacement loading rate controlled at 10 mm/min. The load was gradually increased until irreversible failure of the implant or femur occurred, and the vertical load at failure was recorded.

This was the final destructive test, and a microcomputer-controlled electronic universal testing machine (E45.105; Meters Industrial Systems, USA) was used. The proximal and distal femoral cups were fitted and fixed to the testing machine, and an initial vertical load of 100 N was applied to the proximal femoral cup. The displacement loading rate was controlled at 10 mm/min, and the load was continuously increased until irreversible damage occurred to the femoral model or internal fixation device. The failure mode and final vertical load were subsequently recorded (Figure 6).

### Criteria for experimental evaluation

#### Axial stiffness

Axial stiffness can be described as the extent to which the mechanical parts and components along the axis of the central line resist deformation. It mainly refers to the resistance to tensile deformation. The same size of axial pressure, the greater the stiffness value, which meant that the smaller the deformation, the firmer the implant.

#### Torsional stiffness

When mechanical parts are affected by external force, the ability to resist their own elastic deformation is torsional stiffness. Under the same torsional force, the smaller the torsional angle of implants such as steel plate, the higher the torsional stiffness and the stronger the implant.

#### Axial failure load

The axial failure load refers to the maximum axial load that the structure can withstand when an axial failure occurs under gradually increasing mechanical test conditions in the structural strength test. This load is obtained from the load-displacement curve, and it reflects the maximum load on the implant structure. The greater the axial failure load, the stronger the implant resistance to failure and the better the overall strength.

### Statistical processing

Statistical analysis was performed using SPSS 25.0 statistical software (SPSS Company, USA). A normality test was initially performed, and the measurement data (axial stiffness, torsional stiffness, and axial failure load) conforming to the normal distribution were expressed as mean ± standard deviation. The group design data *t*-test was used for the comparison of bilateral fixation of BCFS and bilateral fixation of LCP + LRP. The two-sided test alpha value was set at <0.05.

## Results

### Axial stiffness

The slope of the curve and the axial stiffness value were obtained using Bluehill 2 software (Figure 4).

When simulating human distal femoral type A3 fractures, the axial stiffness ranged from 1,072.61 ± 113.5 N/mm and 1,184.13 ± 110.24 N/mm in groups A and B, respectively, and there was no significant difference between the two groups (*t* = 1.726, *P* = 0.115 > 0.05, Table 1).

**Table 1:** Comparison between BCFS and LCP + LAP fixation for distal femoral fractures (*x* ± *s*, *n* = 6).

Fixation mode	Axial stiffness (N/mm)	Torsional stiffness (N.m/deg)	Axial failure load (*N*)
BCFS fixation	1,072.61 ± 113.5	3.73 ± 0.12	5,290.45 ± 109.63
LCP + LRP fixation	1,184.13 ± 110.24	3.37 ± 0.04	3,978.43 ± 17.1
*T* value	1.726	6.825	23.28
*P*-value	0.115	<0.001	<0.05

### Torsional stiffness

Torsion angle data were obtained using *P* main software (Figure 5).

When simulating human distal femoral type A3 fractures, the torsional stiffness of groups A and B ranged from 3.73 ± 0.12 N.m/deg and 3.37 ± 0.04 N.m/deg, respectively, and the difference between the two groups was significant (*t* = 6.825, *P* < 0.001, Table 1).

### Axial failure load

In the axial compression failure experiment, with increasing vertical load, the femoral model showed an intertrochanteric fracture, and the model failed (Figure 6).

The axial failure loads of all samples in groups A and B ranged from 5,180.82 to 5,400.08 N and 3,961.33 to 3,995.53 N, respectively. Particularly, the failure loads in group A were higher than those in group B, and the difference was significant (*t* = 23.28, *P* < 0.05, Table 1).

### Performance of damage mode

**Figure 7:**
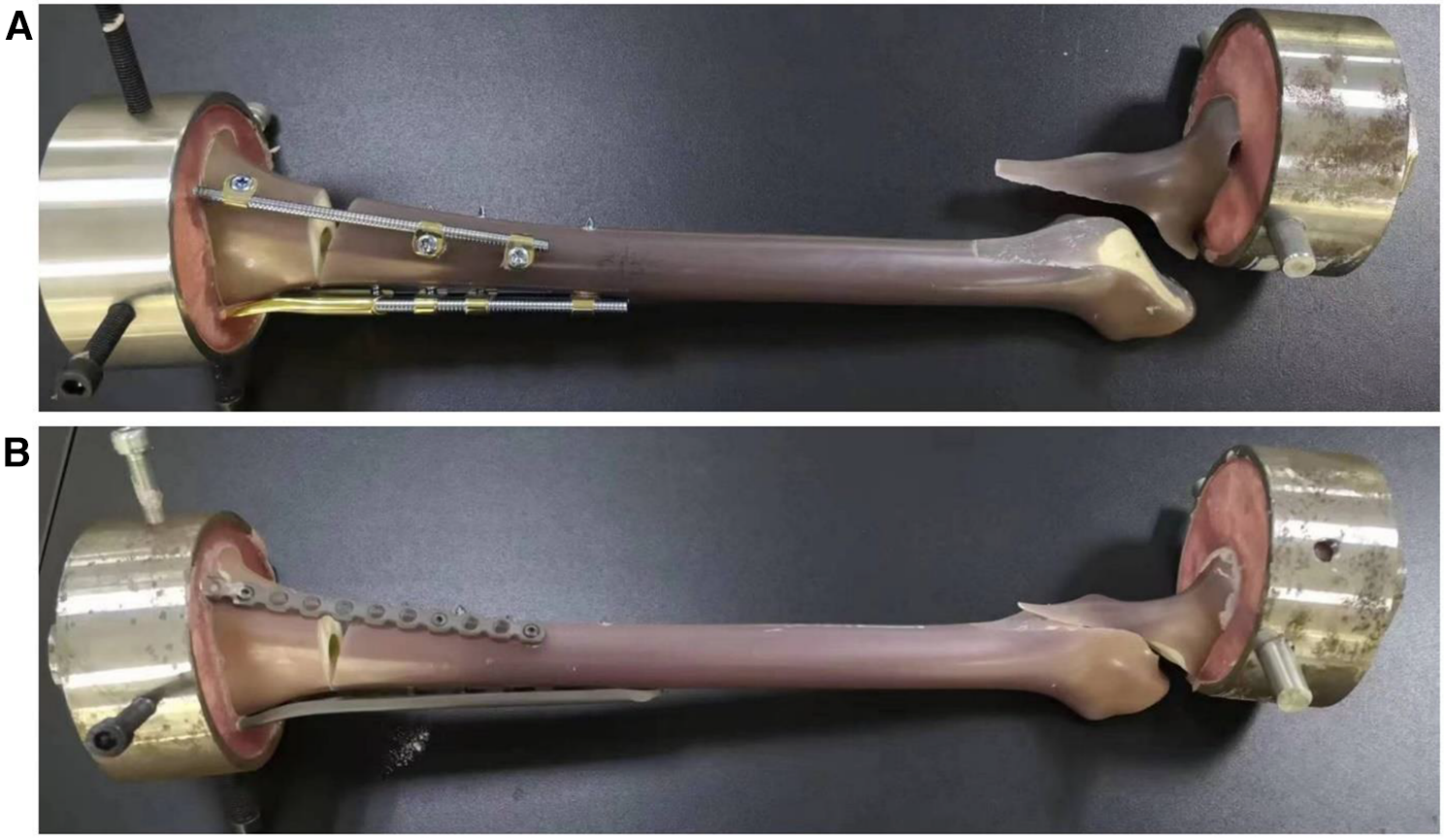
(**A**) BCFS model after axial disruption, (**B**) LCP + LRP model after axial disruption.

After the axial failure load was applied, the fracture models of groups A and B showed an intertrochanteric fracture of the femur. However, no macroscopic deformation of the internal fixation device occurred in groups A and B, and no fracture occurred in the plate, bridging internal fixation system, or corresponding screws (Figure 7).

## Discussion

### Internal fixation methods for type A3 fractures of the distal femur

The treatment of distal femur fractures is a challenge for orthopaedic surgeons. As both periarticular and intra-articular fractures, anatomical reduction and stable fixation of the articular surface are essential for early rehabilitation. Fixation methods for distal femur fractures include extramedullary fixation [locking compression plate (LCP)] and intramedullary fixation (intramedullary nail). Retrograde intramedullary nailing is one of the effective methods for the treatment of distal femoral fractures, and the appropriate retrograde nailing length (27) can be selected according to femoral length and bone morphology. Compared with plate fixation techniques, retrograde nailing techniques require less soft tissue dissection, thereby reducing blood loss (28). Although retrograde nailing offers advantages to patients, various clinical complications associated with retrograde nailing have been reported, such as nonunion, loss of reduction, knee pain, fixation device failure (8). At the same time, the application of extramedullary locking plate has significantly improved the surgical results of middle and distal femoral fractures, so extramedullary locking plate fixation has become the first choice of treatment for distal femoral fractures (12). However, the incidence of adverse complications such as nonunion and fixation failure after internal fixation remains as high as 20% (29). Although anatomical plates of the distal femur solve the problem to some extent, the occurrence of fixation failure remains a problem in complex fracture types. Double plate fixation of the distal femur is initiated to enhance mechanical stability of the construct in high-risk situations. These include (A) different internal fixation methods such as lateral locking compression plate of the distal femur combined with medial reconstruction plate, and (B) lateral application of proximal humerus locking compression plate combined with medial reconstruction plate, and also demonstrate the equivalent effect of the two in distal femoral fractures (30–32). In addition, Wright et al. (33) compared four different internal fixation methods: (1) lateral distal femoral locking plate (DLFLP), (2) retrograde intramedullary nail (rIMN), (3) DLFLP + medial locking compression plate (double-plate construct), and (4) DLFLP + rIMN (plate-nail construct), and concluded that the double-plate device had a stronger fixation effect by biomechanical means. For type A3 distal femoral fractures with medial cortical defects, implant fatigue fractures are likely to occur due to defects in the medial femoral cortex, which leads to insufficient medial cortical support of the distal femur, and long-term bending stresses act on the lateral plate and are more likely to produce complications, such as varus deformity, nonunion, and plate and screw breakage (5, 6). It is more feasible to select double plate as a treatment for distal femoral fractures with medial cortical defects. Studies on comminuted extra-articular fractures (AO Foundation/Orthopaedic Trauma Association type 33A3) provide biomechanical evidence supporting the use of the double plate (33–35). Park et al.'s (34) study showed that additional fixation with a medial plate significantly increased fracture stability under axial loading. However, because double plate fixation and reconstruction of the medial cortex require exposure of the medial distal femur, it leads to greater trauma in these operations and easily causes secondary injury to the knee capsule and vastus intermedius muscle, which aggravates knee capsule and muscle adhesion and is not conducive to postoperative knee function rehabilitation. The presence of these problems limits the application of bilateral plates (36). However, Kang et al. (22) and Wang et al. (23) demonstrated the advantages and clinical effects of BCFS for the above problems, and the results showed that the fractures healed well and functional effects were good.

### Biomechanical differences between the BCFS fixation group and LCP + LRP fixation group in the fixation of AO/OTA type A3 distal femoral fractures

To achieve early functional exercise in the clinical treatment of type A3 distal femoral fractures with medial cortical defects, it is necessary to not only strengthen internal fixation to ensure the stability of the fracture site but also reduce the trauma to the surrounding tissues during surgery. Our study showed no significant difference in the anti-axial compression ability between the two groups when type A3 distal femoral fractures with medial cortical defects were fixed in groups A and B. However, the anti-torsion ability was better in group A than in group B, and the axial failure load in group A was higher than that in group B.

The final failure test showed that the final failure of the model in groups A and B was an intertrochanteric fracture. This mode of failure allows one to speculate that with BCFS and LCP + LRP for fixation of a distal femoral Type A3 fracture model, the overall stress spreads to the femur during axial compression of the femoral model rather than the fixture bearing it independently. Previous biomechanical studies have shown that the femoral shaft of the human body bears a load of approximately one-third of the human body weight in the standing state (37), whereas, in a slow walking state, the femoral shaft bears a load of approximately 2.75 times the body weight owing to increased muscle contraction and joint reaction. Therefore, for a patient weighing between 60 kg and 90 kg, the femoral shaft is expected to be loaded with 200–300 N while standing, contrary to 1,650–2,475 N when walking slowly (38, 39). The axial failure load test results also showed that the failure load of BCFS fixation was 5,290.45 ± 109.63 N and that of LCP + LRP fixation was 3,978.43 ± 17.1 N, both of which exceeded 2,475 N. Thus, theoretically, both internal fixations were sufficient to withstand the load of daily physiological activities. Therefore, it can be inferred from our study that the deformation of internal fixation with BCFS fixation was equivalent to that of the plate system when it resisted axial compression force and that it had better resistance to torsion force. In order to carry out more direct statistical analysis and comparison during the experiment, the range of the two kinds of loads is limited during the experiment, but the set range has included the physiological load approximation at rest, and the results obtained from the axial failure test exceed the maximum load of normal physiological activity.

### Advantages of using the BCFS system for the treatment of type A3 distal femoral fractures with medial cortical defects

As the basic units of the BCFS system, screws, rods, and blocks are combined with single-rod fixation, double-rod fixation, and multiple-rod fixation. Simultaneously, locking screws and non-locking screws can be selected to completely diversify combinations with rods. In addition, a combination of integrated three-dimensional fixation of multiple planes, improved pullout strength, and a larger personalized application space for the treatment of special complex fractures can be created. Some scholars compared the application of the double plate treatment group and unilateral locking plate treatment group in the treatment of comminuted distal femoral fractures and found that the double plate group had fracture healing, whereas two cases in the single plate group had nonunion, and the joint function score gave better results. However, during the actual operation of bilateral plate fixation, finding the bicortical screw path in the condyle of the medial plate was difficult because of the limitation of screw hole position fixation, resulting in difficulty in screw placement. It is noteworthy that according to the specific fixation needs of patients, the BCFS system can be used to design a reasonable combination mode, fixation length, and screw angle to meet the needs of personalized surgery. Secondly, the BCFS internal fixation system is less invasive, as the bridging internal fixation system does not need to be close to the bone surface. In addition, it has little effect on the periosteum and cortical blood supply, and the connecting rod can be shaped according to the anatomical shape of the distal femur, which uses the physiological, anatomical shape, and mechanical mechanics of the remodeled distal femur to ensure the anatomical reduction of the fracture. The position of the connection block is more flexible and can be flexibly adjusted according to the complex situation during the operation; the single-rod single-hole fixation device in the lateral part of the bridging combined internal fixation system at the distal femur can rotate at any angle on the connecting rod to facilitate the search for the best screw channel, unlike the locking plate screw angle fixation. Therefore, the fracture reduction and internal fixation operations are more convenient and flexible. The number and shape of BCFS connecting rods and the type of plate are optional, so theoretically BCFS can meet the treatment requirements for most fractures, such as type A3 fractures of the distal femur in our study. Therefore, this internal fixation system is worthy of being widely popularized and developed in clinical practice. However, surgical techniques and postoperative management vary among surgeons or hospitals. Therefore, adequate surgical skills and experience are necessary during the procedure.

### Limitations of the BCFS system

This study included the following limitations. First, according to a series of experiences in clinical practice in our hospital, this test only compared the traditional LCP + reconstruction plate and bridging plate, and lacked comparison with other surgical methods, and second, the test process was a basic static mechanical test, which could not completely simulate the mechanical state under dynamic conditions; third, a Sawbone model simulating a normal artificial femur was used in this study. Although this model can simulate good bone reserve because type A3 fractures are more likely to occur in elderly patients with osteoporosis, there are still some differences compared to the bone model of osteoporosis. Lastly, this is an *in vitro* test, which cannot simulate the subtle changes in internal fixation existing in the body, and the effects of muscle tissue and vascular nerves cannot be excluded.

## Conclusion

This study showed that when the BCFS system was used to simulate the treatment of type A3 distal femoral fractures with medial cortical defects *in vitro*, its axial compression resistance was not significantly different from that of LCP + LRP fixation, but its torsion resistance was better. Furthermore, both BCFS and LCP + LRP failure loads were greater than those experienced by normal activities. After this test, it can be concluded that BCFS can achieve biomechanically equivalent or even slightly superior results to LCP + LRP (40).

## Data Availability

The original contributions presented in the study are included in the article/Supplementary Material, further inquiries can be directed to the corresponding author.
